# P-1061. *In vitro* Activity of Gepotidacin against *Klebsiella pneumoniae*, Including Molecularly Characterized Fluoroquinolone not Susceptible Subsets Causing Urinary Tract Infections in the United States (2019-2022)

**DOI:** 10.1093/ofid/ofae631.1250

**Published:** 2025-01-29

**Authors:** Rodrigo E Mendes, Danielle Beekman, Cory Hubler, Hank Kimbrough, Renuka Kapoor, Didem Torumkuney, S J Ryan Arends

**Affiliations:** JMI Laboratories, North Liberty, Iowa; Element Materials Technology/Jones Microbiology Institute, North Liberty, Iowa; Element Materials Technology/Jones Microbiology Institute, North Liberty, Iowa; Element Materials Technology/Jones Microbiology Institute, North Liberty, Iowa; GSK, Atlanta, Georgia; GSK, Atlanta, Georgia; JMI Laboratories / Element, North Liberty, Iowa

## Abstract

**Background:**

Gepotidacin (GEP) is a novel, bactericidal, first-in-class triazaacenaphthylene antibiotic that inhibits bacterial DNA replication by a unique mechanism of action, distinct binding site and provides well-balanced inhibition (for most uUTI uropathogens) of two different type II topoisomerase enzymes. This study reports the activity of GEP and other oral antibiotics against *K. pneumoniae*, including molecularly characterized fluoroquinolone (FQ) not susceptible (NS) isolates collected from urinary tract infection (UTI) patients in the US.

**Figure:**
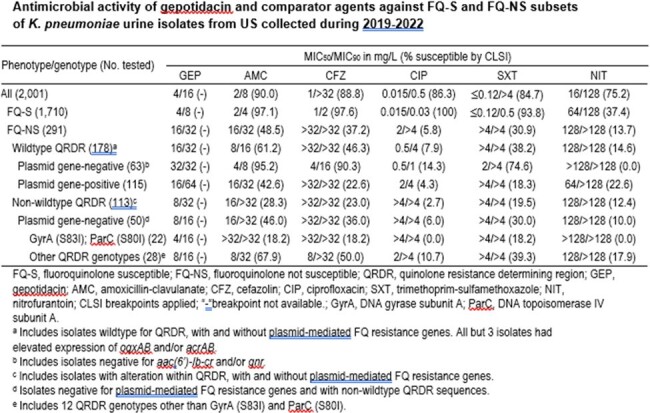

**Methods:**

A total of 2,001 *K. pneumoniae* were collected from 73 sites. CLSI methods were used for both susceptibility (S) testing and MIC interpretation. Isolates with MIC≥0.5 mg/L for ciprofloxacin (NS) and/or ≥1 mg/L for levofloxacin (NS) were screened for FQ-resistance (R) mechanisms (plasmid-mediated quinolone resistance [PMQR]), QRDR mutations, and expression of *acrAB* and *oqxAB* by qRT-PCR.

**Results:**

GEP inhibited 94.9% of all these isolates at ≤16 mg/L (MIC_50/90_ = 4/16mg/L). GEP MIC_50/90_ against FQ-S isolates was 4/8 mg/L, and other agents showed activity against ≥90% of isolates, except nitrofurantoin (37.4%S). 14.5% (291/2,001) of isolates were screened for FQ-R mechanisms. GEP MIC_50/90_ against FQ-NS strains was 16/32 mg/L, whereas other agents had S of 5.8–48.5%. GEP had generally similar activity (MIC_50/90_, 16-32/32-64 mg/L) against isolates with wildtype QRDR and with or without PMQR genes. Most of the isolates (95.2%) without PMQR genes overexpressed *oqxAB* and/or *acrAB*, against which amoxicillin-clavulanate and cefazolin (90.3–95.2%S) were active. GEP had consistent MIC_90_ of 16 mg/L against isolates with distinct QRDR mutations and absence of PMQR genes, whereas other agents had S of ≤68%.

**Conclusion:**

GEP showed activity against FQ-S and FQ-NS *K. pneumoniae* UTI isolates from the US, in particular against FQ-NS isolates carrying QRDR mutations, where standard oral antibiotics showed limited activity. GEP MICs were not substantially affected by QRDR mutations. Highest MICs were seen against FQ-NS isolates with wildtype QRDR and PMQR genes and/or overexpression of efflux-pump genes. These data support the development of GEP for the treatment of uUTI caused by *K. pneumoniae*.

**Disclosures:**

**Rodrigo E. Mendes, PhD**, GSK: Grant/Research Support **Renuka Kapoor, PhD**, GSK: Employee|GSK: Stocks/Bonds (Public Company) **Didem Torumkuney, PhD**, GSK: Employee|GSK: Stocks/Bonds (Public Company)

